# The HTLV-1 Tax interactome

**DOI:** 10.1186/1742-4690-5-76

**Published:** 2008-08-14

**Authors:** Mathieu Boxus, Jean-Claude Twizere, Sébastien Legros, Jean-François Dewulf, Richard Kettmann, Luc Willems

**Affiliations:** 1 University Academia Wallonie-Europe, Molecular and Cellular Biology at FUSAGx, Gembloux, Belgium

## Abstract

The Tax1 oncoprotein encoded by Human T-lymphotropic virus type I is a major determinant of viral persistence and pathogenesis. Tax1 affects a wide variety of cellular signalling pathways leading to transcriptional activation, proliferation and ultimately transformation. To carry out these functions, Tax1 interacts with and modulates activity of a number of cellular proteins. In this review, we summarize the present knowledge of the Tax1 interactome and propose a rationale for the broad range of cellular proteins identified so far.

## 1 Introduction

Human T-lymphotropic viruses (HTLV-1 to -4) belong to the *Deltaretrovirus *genera of the Orthoretrovirinae subfamily. HTLV-1 was the first discovered human retrovirus in the early eighties [1]. HTLV-2 was described two years later [2] whereas HTLV-3 and -4 subtypes were isolated only recently [3,4]. HTLV-1 is the etiological agent of an aggressive leukemia called adult T-cell leukemia/lymphoma (ATL) and a neurodegenerative disease, tropical spastic paraparesis/HTLV associated myelopathy (TSP/HAM). Isolated from a case of hairy-cell leukemia, HTLV-2 is by far less pathogenic although its involvement in the development of TSP has been reported [5,6]. HTLV-3 and -4 have not yet been associated to any pathology, likely due to their recent identification and to the low number of isolates. Three HTLV subtypes have closely related simian viruses (named STLV-1, -2 and -3) while a STLV-5 strain is presently still devoid of a human counterpart [7]. Another related deltaretrovirus, bovine leukemia virus (BLV) is the etiological agent of enzootic bovine leukemia. BLV infection of sheep has been used as an animal model for HTLV [8].

The genome of the HTLV viruses contain typical structural and enzymatic genes (*gag*, *prt*, *pol *and *env*) flanked by two long terminal repeats (LTRs) but also harbors an additional region called pX located between the *env *gene and the 3'-LTR. This region contains at least four partially overlapping reading frames (ORFs) encoding accessory proteins (p12^I^, p13/p30^II^), the Rex post-transcriptional regulator (ORF III) and the Tax protein (ORF IV). The complementary strand of the HTLV-1 proviral genome is also transcribed, yielding spliced isoforms of the Hbz factor [9-11]. Hbz interacts with factors JunB, JunD, CREB and CBP/p300 to modulate gene transcription [12-14]. There is an inverse relantionship between high Hbz and low Tax expresssion in primary ATL [15].

Among proteins encoded by HTLV-1, Tax1 exerts an essential role in viral transcription as well as in cell transformation [11,16-18]. These pleiotropic functions are directed by a very wide spectrum of interactions with cellular proteins. In this review, we summarize the current knowledge pertaining to the Tax1 interactome and focus more particularly on its impact on transcription, viral persistence and transformation.

## 2 Interaction of Tax1 with transcription factors and post-transcriptional regulators

In eukaryotes, initiation and elongation of gene transcription requires decondensation of the locus, nucleosome remodeling, histone modifications, binding of transcriptional activators and coactivators to enhancers and promoters and recruitment of the basal transcription machinery to the core promoter [19,20]. Tax1 is a pleiotropic transcription factor that interferes with several of these mechanisms and modulates transcription of a wide range of cellular genes. In fact, Tax1 deregulates expression of more than one hundred genes [21] through interactions with transcriptional activators, basal transcription factors and proteins involved in chromatin remodeling. Moreover, Tax1 associates with proteins involved post-transcriptionnal control of mRNAs and further modulates gene expression.

### 2.1 Transcriptional activators and repressors

#### 2.1.1 CREB/ATF factors

Tax1 was initially described as an activator of LTR-directed transcription [22]. Three imperfectly conserved 21-base-pair (bp) repeat sequences called (TxRE) located in the U_3 _region of the LTR are required and sufficient to confer Tax1 responsiveness [23]. The TxRE element contains an octamer motif TGACG(T/A)(C/G)(T/A) that is flanked by a G stretch and a C stretch at the 5' and 3' sides, respectively [24]. Interestingly this octamer shares homology with the consensus cAMP-responsive element (CRE) 5'-TGACGTCA-3' [24]. Nevertheless, Tax1 exhibits poor affinity for DNA and does not bind directly to the TxRE element [25] but interacts with CRE-binding/activating transcription factors (CREB/ATF). In fact, Tax1 interacts *in vitro *with a number of proteins of the CREB/ATF family of transcription factors: CREB, CREM, ATF1, ATF2, ATF3, ATF4 (CREB2) and XBP1 (X-box-binding protein 1) [26-31]. These proteins share a common cluster of basic residues allowing DNA binding and a leucine zipper (b-Zip) domain involved in homo- and heterodimerization. Dimer formation modulates their DNA binding specificity and transcriptional activity [32]. Biochemical studies revealed that Tax1 promotes formation of a Tax1-CREB/ATF-TxRE ternary complex *in vitro *by interacting with the b-Zip domain of CREB/ATF factors. Mechanistically, Tax1 enhances the dimerization of CREB/ATF factors, increases their affinity for the viral CRE [33-36] and further stabilizes the ternary complex through direct contact of the GC-rich flanking sequences [37,38]. Tax1 then recruits co-activators (CBP/p300 and P/CAF) to facilitate transcriptional initiation (see 2.3.1). The ability of Tax1 to dimerize is required for efficient ternary complex formation and for optimal transactivation [39,40]. Interaction of Tax1-CREB/ATF with the LTR promoter DNA was further explored by chromatin immunoprecipitation (ChIP) [41]. In HTLV-1 infected human T-cells (SLB-1), Tax1 and a plethora of CREB/ATF factors as well as other b-Zip proteins bind to the LTR promoter, further confirming interaction *in vivo*. The fact that Tax1 interacts with ATFx adds another level of complexity since this factor represses Tax1-mediated LTR activation [42]. Tax1 is thus able to interact with positive as well as with negative CREB/ATF factors to modulate LTR promoter-directed activity.

Tax1 also binds to CREB co-activator proteins called transducers of regulated CREB activity (TORCs). In fact, Tax1 interacts with the three members of this family (TORC1, TORC2 and TORC3) [43,44] and TORCs cooperate with Tax1 to activate the LTR in a CREB and p300-dependent manner. Thus, TORCs are thought to associate with the Tax1 ternary complex and participate to transcriptional activation.

CREB/ATF members play a role in cell growth, survival and apoptosis by regulating CRE-directed gene transcription in response to environmental signals such as growth factors or stress [32,45]. Furthermore, CREB/ATF proteins also have significant impact on cancer development [45]. Depending on the cell type, Tax1 mutants deficient for CREB activation are incompetent for transformation or induction of aneuploidy [46-50]. Tax1 activates a variety of cellular genes through its interactions with CREB/ATF proteins, for example those encoding interleukin 17 or c-fos [51,52]. Conversely, Tax1 also represses expression of genes like cyclin A, p53 and c-myb by targeting CREB/ATF factors [53-55]. Transcriptomic profiling of cells expressing either a wild-type or a CREB-deficient Tax1 protein revealed several cellular genes controlled by CRE elements activated by Tax1 [50]. Among these, Sgt1 (suppressor of G2 allele of SKP1) and p97(Vcp) (valosin containing protein) have functions in spindle formation and disassembly, respectively.

Together, these reports thus demonstrate that Tax1 interacts with a series of CREB/ATF factors and modulates expression of viral and cellular genes through CRE elements. The specific contribution of each CREB/ATF member in Tax1-mediated gene transcription remains unclear.

#### 2.1.2 Serum responsive factor and members of the ternary complex factor

HTLV-1 infected T-cell lines expressing Tax1 display increased expression of AP1 (activator protein 1), a homo- or heterodimeric complex of Fos (c-Fos, FosB, Fra1 and Fra2) and Jun (c-Jun, JunB and JunD) [56,57]. Fos and Jun are under the transcriptional control of the serum responsive factor (SRF) in response to various stimuli such as cytokines, growth factors, stress signals and oncogenes. SRF binds to the SRF responsive element (SRE) located in the Fos/Jun promoters which contains two binding sites: a CarG box (CC(A/T)_6_GG) and an upstream Ets box (GGA(A/T)). Once SRF occupies the CArG box, the ternary complex factor (TCF) establishes protein interaction with SRF and subsequently binds the upstream Ets site. This complex then recruits the co-activators P/CAF and CBP/p300 to activate transcription.

In reporter assays, Tax1 activates transcription of promoters under the control of SRE motifs [52,56,58] without direct binding to the DNA but through interactions with transcription factors associated with the SRF pathway. Tax1 has been shown to bind directly to SRF [59-61] and to various members of the TCF complex such as Sap1 (SRF accessory protein 1), Elk1, Spi1 (spleen focus forming virus (SFFV) proviral integration oncogene 1) and Ets1 [49,62,63]. Tax1 interaction with SRF results in increased binding of SRF to the SRE and altered site selection [64]. Once the complexes are stabilized, Tax1 recruits the co-activators CBP/p300 and P/CAF (see 2.3.1) and mediates transactivation [63].

It thus appears that Tax1 activates transcription from CREB- and SRF-responsive sites through a similar mechanism which involves its interaction with transcription factors resulting in enhanced DNA binding, altered site selection and coactivator recruitment [16].

#### 2.1.3 Nuclear factors κB (NF-κB)

HTLV-1 infected cells display increased expression of various cytokines and cytokine receptors such as interleukin 2 (IL2) and the α-subunit of its high-affinity receptor complex (IL2Rα) [65-68]. Induction of IL2 and IL2Rα expression is mediated by Tax1 activation of the NF-κB/Rel family of transcription factors [69,70]. By modulating expression of a wide range of genes involved in apoptosis, proliferation, immune response and inflammation, NF-κB is thought playing a central role in Tax1-mediated cell transformation [16].

In mammals, the NF-κB family of transcription factors is composed of five structurally related members, RelA, RelB (p65), c-Rel, NF-κB1 (p50/p105) and NF-κB2 (p52/p100) which form various dimeric complexes that transactivate or repress target genes bearing a κB enhancer [71,72]. p105 and p100 are precursor proteins that are processed proteolytically to the mature p50 and p52 forms, respectively. These factors share a common Rel-homology domain (RHD) mediating their dimerization, DNA binding and nuclear localization. In non-activated cells, NF-κB dimers are trapped in the cytoplasm by inhibitory proteins called IκBs such as p105, p100, IκBα, IκBβ and IκBγ (C-terminal region of p105), that mask the nuclear localization signal of NF-κB factors through physical interaction [71,72]. NF-κB activation involves phosphorylation of IκB inhibitors by the IκB kinase (IKK), which triggers their ubiquitination and subsequent proteasomal degradation, resulting in nuclear translocation of NF-κB dimers [72,73].

Tax1 associates with RelA, c-Rel, p50 and p52 after their translocation in the nucleus [61,74,75] but also directly recruits RelA from the cytoplasm [76,77]. After interaction with these NF-κB factors, Tax1 increases their dimerization which is essential for binding to target promoters [61,75,78]. When the complex is bound to the promoter, Tax1 recruits the CBP/p300 and PCAF co-activators [79,80], leading to transcriptional activation

#### 2.1.4 Other transcription factors

Tax1 has been shown to associate with CCAAT binding proteins such as NF-YB (nuclear factor YB subunit) and C/EBPβ (CCAAT/enhancer-binding protein β) [81-83]. Through its binding to NF-YB, Tax1 activates the major histocompatibility complex class II promoter [82]. Besides, C/EBPβ acts as a transcriptional repressor by preventing Tax1 binding to the LTR [83]. On the other hand, Tax1 increases binding of C/EBPβ to and activates the IL-1β promoter [81]. It is noteworthy that C/EBP factors have been implicated in regulation of cellular proliferation and differentiation but also in tumor formation and leukemia development [84].

Tax1 forms ternary complexes *in vitro *with Sp1 (specificity protein 1)/Egr1 (early growth response 1) [85] and Sp1/Ets1 [62], thereby participating directly in transcriptional activation of the c-sis/PDGF-B (platelet-derived growth factor B) proto-oncogene and PTHrP (parathyroid hormone-related protein) P2 promoters, respectively. Of note, PTHrP is up-regulated during immortalization of T-lymphocytes by HTLV-1 and plays a primary role in the development of humoral hypercalcemia of malignancy that occurs in the majority of patients with ATL [86,87].

Tax1 further associates with nuclear respiratory factor 1 (NRF1) and activates the CXCR4 chemokine receptor promoter [88].

Finally, the transcriptional repressor MSX2 (msh homeobox homolog 2) impairs Tax1 mediated transactivation through direct binding [89].

### 2.2 Basal transcription factors

Tax1 interacts with TF_II_A (transcription factor II A) and with two subunits of TF_II_D: TBP (TATAA-binding protein) and TAF_II_28 (TBP-associated factor II 28) [90-92]. These basal transcription factors compose the preinitiation transcription complex responsible for the recruitment of RNA polymerase II. Owing to this interaction, Tax1 increases the binding of TBP to the TATAA site and further stimulates transcription initiation from the LTR [93].

### 2.3 Chromatin modifying enzymes

Structural variations of chromatin range from condensed heterochromatin to more open euchromatin, a process that depends on antagonistic effects between multiple protein complexes. Among the complexes affecting chromatin structure, there are those who are capable of altering the histones themselves, the histone deacetylases (HDAC), acetyltransferases (HAT), demethylases (HDM) and methyltransferases (HMT), and those that use the energy of ATP to change the structure of the nucleosome as the SWI/SNF complex [94-96]. Tax1 expression and HTLV-1 infection both reduce histone levels in T cells [97]. Moreover, Tax1 interacts directly and recruits several proteins involved in chromatin remodeling to modulate gene transcription. The involvement of Tax1-binding proteins in transcriptional activation has been primarily described in the context of the viral LTR. Nevertheless, similar mechanisms are also likely to participate in the activation of cellular promoters.

#### 2.3.1 HATs

Acetylation of lysine residues located in the N-terminal tails of histone proteins by HATs is a crucial step for activation of gene transcription. Tax1 interacts with several HATs: p300, its homologous CREB binding protein (CBP) and p300/CBP associated factor (P/CAF) [98-102]. Tax1 recruits the CBP/p300 and P/CAF once the Tax1-CREB-TxRE complex is stabilized (see 2.1.1), each of which being able to enhance Tax1-mediated transactivation of a transiently transfected LTR reporter. CBP/p300 and P/CAF bind independently on different regions of Tax1 and interaction of Tax1 with these two cofactors is required for optimal transcriptional activity from transiently transfected but also stably integrated LTR reporters [101-103]. Surprisingly, P/CAF but not CBP/p300 is able to enhance transcription from the LTR independently of its HAT activity [101,103]. Tax1 mediates recruitment of CBP/p300 on reconstituted chromatin templates and facilitates transactivation in a HAT-activity dependent manner [104,105]. CBP/p300 presence at the LTR template correlates with histone H3 and H4 acetylation as well as increased binding of basal transcription factors and RNA polymerase II. ChIP analyses with HTLV-1 infected T cell lines indicate that Tax1, CBP/p300 and acetylated histone H3 and H4 are indeed associated with the LTR promoter [41,105].

There is a long lasting debate about how Tax1 recruits CBP/p300 at the Tax1-CREB/ATF-TxRE complex. Phosphorylation of CREB at serine 133 by protein kinases A or C is required for CBP/p300 recruitment via physical interaction with the KIX domain [106-108]. It has long been suggested that Tax1 bypasses the requirement for CREB phosphorylation to recruit coactivators [98,100]. Nevertheless, recent reports indicate that Tax1 rather cooperates with phosphorylated CREB (pCREB) to induce transactivation [109,110]. High levels of pCREB are detected in Tax1 expressing cells and in HTLV-1-infected human T-lymphocytes [110]. Tax1 and pCREB interact simultaneously at two distinct binding sites on the KIX domain forming a very stable complex with the viral CRE [110,111]. Both CREB phosphorylation and Tax1 binding are needed for efficient interaction of full-length CBP to pCREB and subsequent transcriptional activation [112].

Finally, Tax1 is able to repress the activity of some transcription factors by competitive usage of CBP, p300 and P/CAF. As mentionned above, stable complex formation between Tax1, a transcription factor (e.g. CREB or SRF) and CBP/p300 contributes to transcriptional activation. On the contrary, when Tax1 has poor affinity for a transcription factor (e.g. p53, MyoD or STAT2), it interferes with co-activator recruitment and prevents their activition [113-116]. Although controversial, this mechanism termed trans-repression could partipate to p53 inactivation in Tax1 expressing cells and HTLV-1 infected lymphocytes (for a review see [117]).

#### 2.3.2 HDACs

Among three HDACs (-1, -2 and -3) interacting with the viral LTR in HTLV infected cell lines [118], Tax1 binds directly to HDAC1. HDAC1 overexpression represses Tax1-mediated transactivation owing to its HDAC activity [119]. Nevertheless, the presence of Tax1 and HDAC1 on the viral promoter is mutually exclusive [118,120]. HDAC1 binds to the non-activated LTR and is released from the promoter through physical interaction with Tax1 allowing recruitment of co-activators and transcription initiation. Tax1 is also able to tether HDAC1 to the tyrosine phosphatase SHP1 promoter and selectively down-regulate gene expression [121].

HDACs form multiprotein complexes together with DNA-histone binding proteins such as SMRT (silencing mediator for retinoid and thyroid receptor) and MBD2 (methyl-CpG-binding domain 2) that both interact with Tax1 and are involved in Tax1 transcriptional activities [122,123]. It thus seems that Tax1, through direct association with HDACs and HDAC-containing complexes is able to selectively activate or repress viral and cellular genes expression.

#### 2.3.3 HMTs and HDMs

Mono-, di- and tri-methylation of histone H3 at lysine 9 (H3K9) play a crucial role in structural modification of chromatin. Tax1 associates with two enzymes involved in regulation of H3K9 methylation: SUV39H1 (suppressor of variegation 3–9 homologue 1), a HMT and JMJD2A (Jumonji containing domain 2A), a HDM [124,125]. Methylated H3K9 is a hallmark of transcriptionally inactive chromatin whereas demethylation rather promotes transcriptional activation [126]. SUV39H1 interacts with Tax1 and represses Tax1-mediated transactivation of the LTR [124]. JMJD2A is highly expressed in HTLV-1 infected cell lines but its role on Tax1-mediated transcription is currently unknown [125].

Methylation of histone H3 at arginine residues is another important regulatory mechanism of transcriptionnal regulation. Tax1 associates with coactivator-associated arginine methyltransferase (CARM1), which preferentially induces methylation at residues R2, R17 and R26 of histone H3 [127]. CARM1 is recruited by Tax1 to the LTR and increases Tax1-mediated transactivation of the LTR. Consistently, silencing of CARM1 impairs Tax1 transcriptional activation, R2-, R17- and R26-methylated histone H3 proteins being present on the LTR promoter in HTLV-1 infected cells.

Tax1 thus interacts with different histone methyltranferases and demethylases to modulate histone methylation and regulate gene expression.

#### 2.3.4 The SWI/SNF complex

The SWI/SNF (Switch/Sucrose Non Fermentable) complex utilizes the energy of ATP hydrolysis to remodel chromatin structures, thereby allowing transcription factors to gain access to DNA during initiation and elongation steps of transcription [128,129]. Tax1 interacts with different components of SWI/SNF: BRG1, BAFs 53, 57 and 155 [130]. Overexpression and silencing of BRG1 increments and impedes Tax1 transactivation of the LTR, respectively [130]. It was first suggested that Tax1 targets BRG1/BRM downstream of RNA polymerase II in order to prevent stalling of transcription. This model was apparently contradicted by the capacity of Tax1 to efficiently activate transcription from chromosomally integrated LTR and NF-κB promoter in a BRG1/BRM deficient cell line [131]. Nevertheless, this observation does not exclude that factors of the SWI/SNF complex cooperate with Tax1 to promote gene transcription. Consistent with this idea, Tax1 cooperates with SWI/SNF complex and RNA polymerase II to promote nucleosome eviction during transactivation [132]. Histone eviction increases accessibility of DNA to transcription factors and requires activity of SWI/SNF and RNA polymerase II [128,133]. Of note, Tax1 may also impact indirectly on SWI/SNF function [134] by interaction with DNA topoisomerase I [135].

Tax1 is thus able to target SWI/SNF complex components to promote nucleosome displacement and participate to transcriptional activation.

### 2.4 Positive transcription elongation factor b (P-TEFb) and sc35

The switch from initiation of transcription to elongation requires promoter clearance and phosphorylation of the RNA polymerase II carboxyl-terminal domain (CTD) [19]. Phosphorylaton of CTD on serine 5 (S5) and 2 (S2) requires the kinase activities of the basal transcription factor TF_II_H and CDK9, respectively. In the cell, CDK9 together with regulatory subunits cyclin T1, -T2, or -K compose the positive transcription elongation factor b (P-TEFb) that ensures the elongation phase of transcription by RNA polymerase II [136,137]. Tax1 recruits P-TEFb to the viral promoter by interacting with cyclin T1 and CDK9 silencing or depletion inhibits Tax1-mediated transactivation [138,139]. In fact, recruitment of P-TEFb activity to the LTR promoter increases CTD phosphorylation at serine S2 (but not S5) and allows transcriptional activation [138].

Recent data suggest that the splicing factor sc35 has a critical role in P-TEFb recruitment and positively impacts on transcription [140]. Tax1 binds and colocalizes with sc35 and P-TEFb in nuclear transcriptional hot spots termed speckled structures [141].

### 2.5 Nuclear receptors

Nuclear receptors (NR) belong to a large family of ligand-activated transcription factors that regulate gene expression in response to steroids, retinoids, and other signaling molecules [142]. Tax1 functions as a general transcriptional repressor of nuclear receptors such as glucocorticoid receptors (GR) [143]. A Tax1-binding protein referred to as Tax1BP1 and identified in a yeast two hybrid screen acts as a transcriptional co-activator for NR. Tax1 represses GR signaling by dissociating Tax1BP1 from the receptor-protein containing complex. Consistently, Tax1BP1 overexpression restores GR signaling in Tax1-expressing cells [144].

### 2.6 Post-transcriptional and translational regulators

Tax1-directed gene expression is further regulated at the post-transcriptional and translational levels through protein-protein interactions. Among these, Tax1 associates with TTP, Int6 and TRBP.

#### 2.6.1 Tristetraprolin (TTP)

TTP belongs to a family of adenine/uridine-rich element (ARE)-binding proteins that contain tandem CCCH zinc finger RNA-binding domains [145]. TTP is therefore an important player in posttranscriptional regulation of mRNA containing ARE elements. Indeed, TTP delivers ARE-containing mRNAs in discrete cytoplasmic regions, called RNA granules, involved in regulation of translation or decay of these transcripts [146]. The repertoire of ARE-containing genes includes Tumor Necrosis Factor α (TNFα) and Granulocyte Macrophage-Colony-Stimulating Factor (GM-CSF) [145] involved in cell signaling, metabolism, cell proliferation, immune response, death, differentiation and morphogenesis [147].

Tax1 interacts with TTP and redirects TTP from the cytoplasm to the nuclear compartment as well as in a region surrounding the nucleus [148]. Through its interactions with TTP, Tax1 stabilizes TNFα mRNA and indirectly increases TNFα protein expression. This observation is of importance for the cell transformation process induced by HTLV-1, because TNFα overexpression plays a central role in pathogenesis.

#### 2.6.2 Int6 and TRBP

Tax also binds Int6 (Integration site 6) and TRBP (TAR binding protein) that regulate translation and RNA interference, respectively. In fact, Int6 is a subunit of translation initiation factor eIF3, which regulates mRNA binding to the ribosome [149] while TRBP (TAR binding protein) is a componant of RISC (RNA-induced silencing complex) that mediates RNA interference [150]. Currently, the role of these interactions remains unclear.

### 2.7 A global model of Tax1 transactivation

**Figure 1:**
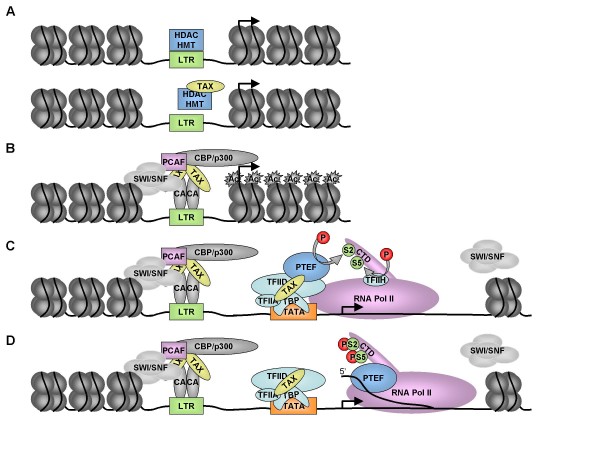
**Global model of Tax1 mediated transactivation**. Tax1 relieves transcriptional repression of the LTR through direct interaction with HDAC (i.e. HDAC1) and/or HMT (panel A). Tax1 recruits CREB/ATF transcription factors (CA in panel B), histone modifying enzymes and chromatin remodelers (SWI/SNF, P/CAF and CBP/p300). Tax1 then allows binding of basal transcription factors on the TATA box (panel C). Once the initiation complex is formed, Tax1 recruits the P-TEFb factor, leading to CTD phosphorylation and processive elongation (panel D). Finally, interaction of Tax1 with SWI/SNF prevents stalling of transcription elongation. Adapted from [120,132,138,316].

Most of the data summarized in the former paragraphs relate to transcriptional activation of the LTR by Tax1 although it is likely that similar mechanisms also pertain to cellular promoters. Figure 1 recapitulates the mechanisms of transactivation: Tax1 relieves transcriptional repression through direct interaction with HDAC (i.e. HDAC1) and/or HMT (panel A). Tax1 interacts with CREB/ATF factors (CA) and enhances their binding to the LTR (panel B). When complexes are stabilized on the promoter, Tax1 recruits histone modifying enzymes and chromatin remodelers. This step affects chromatin structure and allows binding of basal transcription factors on the TATA box that is further stabilized by Tax1 interaction with TF_II_A, TF_II_D and TBP (panel C). Once the initiation complex is formed, Tax1 recruits the P-TEFb factor, leading to CTD phosphorylation and processive elongation (panel D). Finally, interaction of Tax1 with SWI/SNF prevents stalling of transcription elongation.

## 3 Tax1 interaction with proteins involved in cell signaling

### 3.1 NF-κB signaling

NF-κB can be activated by a series of stimuli such as antigens or cytokines that trigger two alternative pathways (so-called canonical and non-canonical). The canonical pathway is engaged in response to inflammatory stimuli (such as TNF-α and interleukin 1 IL-1), T-cell receptor activation or exposure to lipopolysaccharide (LPS). This pathway begins with the phosphorylation of IκB inhibitors by the IκB kinase (IKK), a complex of IKKα, IKKβ and IKKγ/NEMO (NF-κB Essential Modulator). IKK is activated by a mitogen-activated protein kinase kinase kinase (MAP3K) that phosphorylates the IKKα and IKKβ subunits. Phosphorylation of IκB inhibitors triggers their ubiquitination and subsequent degradation by the 26S proteasome, resulting in nuclear translocation of NF-κB dimers (e.g. p50/relA) [72,73]. The non-canonical pathway, which can be induced by stimuli such as CD40 ligand, involves IKKα activation upon phosphorylation by NF-κB inducing kinase (NIK). IKKα then phosphorylates the C-terminal region of p100 leading to subsequent processing of the p100/RelB complex into p52/RelB and its translocation into the nucleus [151]. Interestingly, p52/RelB and p50/RelA dimers target distinct κβ enhancers thereby activating different gene subsets.

Tax1 stimulates both canonical and non-canonical pathways and constitutively activates NF-κB in HTLV-1 infected cells [152-154]. The above mentionned interactions of Tax1 with NF-κB transcription factors (see 2.1.3) only explains part of Tax1-mediated NF-κB activation since this completion of this process also requires cytoplamic events. In the canonical pathway, Tax1 associates with the IKKγ/NEMO subunit [155,156] as well as with activating upstream kinases such as MAPK/ERK kinase kinase 1 (MEKK1) and TGF-β activating kinase 1 (TAK1) [157,158] (see 3.2). Tax1 thus connects activated kinases to the IKK complex and forces the phosphorylation of IKKα and IKKβ leading to degradation of IκBα and IκBβ [155,156]. In addition, Tax1 binds directly to the IKKα and IKKβ subunits and activates their kinase activity independently of the upstream kinases [159]. Consistently, silencing of MEKK1 and TAK1 does not impair Tax1-induced NF-κB activation [160]. A third level of Tax1 interference with the canonical pathway is its direct binding to IκBs and their degradation independently of IKK phosphorylation [161,162]. Tax1 further interacts two subunits of the 20S proteasome (HsN3 and HC9), favors anchorage of p105 and accelerates its proteolysis [163]. Tax1 thus leads to IκB degradation at multiple levels, thereby allowing nuclear translocation of NF-κB independently of external stimuli. Besides, activation of the non-canonical pathway by Tax1 requires its interaction with IKKγ and p100 [152,154]. Through these interactions, Tax1 targets IKKα to p100, induces p100 processing and nuclear translocation of the p52/RelB dimer. It thus appears that IKKγ is an important Tax1 docking site for activation of both pathways.

Post-translationnal modifications of IKKγ such as phosphorylation and K63 ubiquitination fine-tune NF-κB signaling [164,165] and are modulated by Tax1 through complex formation. In fact, PP2A activates the IKK complex by promoting dephosphorylation of IKKγ serine 68 [166,167]. Tax1 complexes with PP2A and IKKγ, maintaining the IKK complex in an active state that is required for activating NF-κB [168,169]. Ubiquitination is targeted by Tax1 through interaction with Ubc13 and Tax1BP1 [170,171]. Ubc13, an E2 ubiquitin-conjugating enzyme, is required for Tax1 interaction with IKKγ and subsequent NF-κB activation. Tax1BP1 participates to the formation of an ubiquitin-editing complex together with the deubiquitin enzyme (DUB) A20 and plays a pivotal role in termination of NF-κB and JNK signaling by regulating the activity of A20 [171-173]. A20 inhibits IKK activation by cleaving K63 linked polyubiquitin chains on tumor necrosis factor receptor (TNFR) signaling-associated factor 6 (TRAF6), receptor interacting protein 1 (RIP1) and IKKγ [174]. By disruption of complex A20-Tax1BP1, Tax1 inactivates DUB function of A20 and prevents downregulation of IKKγ ubiquitination. Consistent with this model, IKKγ is ubiquitinated in Tax1-expressing cells and in a series of HTLV-1 infected cell lines [160,171] providing a rationale for the constitutive activation of NF-κB pathway.

### 3.2 Mitogen-activated kinases (MAPKs)

MAPKs are serine/threonine-specific protein kinases that respond to external mitogen stimuli such as growth factors, cytokines or physical stress. MAPK signaling relies on a sequential phosphorylation cascade that goes through MAP kinase kinase kinase (MAP3K) to MAP kinase kinase (MAP2K) and finally to MAPK. The MAPK family includes the extracellular signal-regulated kinase protein homologues 1 and 2 (ERK1/2), ERK5, the c-Jun N-terminal Kinase 1, 2 and 3 (JNK1/2/3) also known as stress-activated protein kinase-1 (SAPK-1), the p38 isoforms (p38α/β/δ), ERK6, ERK3/4 and ERK7/8 [175]. Tax1 interacts with two MAP3Ks: MEKK1 and TAK1 [157,158].

#### 3.2.1 MEKK1

MEKK1 primarily regulates JNK and ERK1/2 but also contributes to the NF-κB pathway [176,177]. Tax1 binds to the amino terminus of MEKK1 and stimulates MEKK1 kinase activity [157]. As a result, Tax1 expression increases IKKβ activity, leading to phosphorylation and degradation of IκBα. Dominant negative mutants of both IKKβ and MEKK1 prevent Tax1 activation of the NFκB pathway but, intriguingly, silencing of MEKK1 does not affect Tax1-induced NF-κB activation [160].

#### 3.2.2 TAK1

TAK1 is involved in JNK, TGF-β and NF-κB dependent signaling pathways [178]. TAK1 acts in concert with TAK1 binding proteins (TABs) which link TAK1 to the upstream activating TNF receptor associated factor (TRAFs) proteins. TAK1 phosphorylates IKKβ and MKK6, thereby activating NF-κB and JNK [179].

TAK1 is constitutively activated in Tax1-expressing cells and in HTLV-1 infected lymphocytes [158,160,180]. Tax1 activates TAK1 through complexation with TAK1 and TAB2 and connects TAK1 onto the IKK complex thereby stimulating IKK activity [180,181]. Consistently, overexpression of TAK1 or TAB2 increases Tax1 transactivation of a NF-κB reporter [180,181]. However, RNA interference of TAK1 suppresses activation of JNK and p38 but not NF-κB. Constitutive activation of TAK1 is thus not absolutely required for NF-κB activation [160,180]. TAK1 rather participates to JNK signaling, which is constitutively activated in Tax1-expressing cells, in Tax1-transformed murine fibroblasts and in human lymphocytes transformed with HTLV-1 [182-185].

### 3.3 GPS-2

By linking the nuclear co-receptor (NCoR)-HDAC3 complex to intracellular JNK signaling, G protein pathway suppressor 2 (GPS2) suppresses Ras/MAPK signaling and JNK1 activation [183,186,187]. Indeed, the NCoR-HDAC3 deacetylase activity represses transcription of genes involved in JNK signaling [187]. Through interaction with GPS2, Tax1 potently inhibits GPS2-mediated inactivation of JNK signaling [183]. Tax1 thus targets multiple proteins (i.e. TAK1 and GPS2) to constitutively activate JNK signaling.

### 3.4 GTP-binding proteins

The guanine nucleotide-binding proteins GTPases are molecular switches that cycle between active (GTP-bound) and inactive (GDP-bound) states. The G protein family includes Ras-related GTPases (or small GTPases) and heterotrimeric G proteins (α, β and γ subunits) that are activated by G protein-coupled receptors.

#### 3.4.1 Rho GTPases and the cytoskeleton proteins

Tax1 complexes with several members of the small GTPase Rho family such as RhoA, Rac, Gap1m and Cdc42 [130]. Rho GTPases are activated in response to external stimuli (e.g. growth factor, stress, cytokines) and exert a wide range of biochemical functions like cytoskeleton organization, regulation of enzymatic activities as well as gene expression [188]. Notably, Tax1 binds to proteins involved in cytoskeleton structure and dynamics: α-internexin, cytokeratin, actin, gelsolin, annexin and γ-tubulin [130,189-191]. Through these interactions, Tax1 might thus connect Rho GTPases to their targets and affect cytoskeleton organization. Consistent with this idea, Tax1 localizes around the microtubule organization center (MTOC) and in the cell-cell contact region [192]. Thereby, Tax1 provides an intracellular signal that synergizes with ICAM1 engagement to cause the T-cell microtubule polarization and formation of the virological synapse. Through the formation of complexes with both Rho GTPases and their targets, Tax1 could thus favor HTLV-1 cell-to-cell transmission.

Since Rho GTPases modulate a wide range of signaling networks (SRF, JNK, p38 and NF-kB) [188], complex formation with Tax1 is also likely to modulate transcription.

#### 3.4.2 Heterotrimeric Gβ subunit

Heterotrimeric G proteins are the molecular switches that turn on intracellular signaling cascades in response to activation of G protein coupled receptor (GPCR). After binding of an agonist, the activated GPCR induces an exchange of GDP to GTP on the Gα subunit and facilitates the dissociation of GTP-bound Gβγ and Gα subunits [193]. Through its interaction with Gβ, Tax1 affects SDF-1 dependent activation of the CXCR4 GPCR chemokine receptor. Tax1 enhances response to SDF-1 resulting in MAPK pathway over-activation and increased cell chemotaxis. The HTLV-1 associated pathologies (ATL, HAM/TSP and dermatitis) are characterized by invasion and accumulation of infected T-cells in organs such as lymph nodes, central nervous system or dermis [194]. These results thus provide a rationale for the mechanisms of cell migration observed in HTLV-1 associated pathologies.

### 3.5 Phosphatidylinositol 3-kinase and AP-1

Phosphatidylinositol 3-kinase (PI3K) and its downstream effector Akt play a pivotal role in regulation of nutrient metabolism, cell survival, motility, proliferation and apoptosis. The PI3K family comprises eight members divided into three classes according to their sequence homology and substrate preference [195,196]. PI3K activation results in phosphorylation of Akt at Ser^473 ^which in turn triggers a broad range of regulatory proteins and transcription factors like AP1 [197].

PI3K-Akt is activated in Tax1-transformed murine fibroblasts and is required for cell transformation [198]. Tax1 complexes with the p85α regulatory subunit of PI3K [199] and inhibits activity of the p110α catalytic protein. p85α/p110α belong to the class IA PI3Ks and are activated by receptor tyrosine kinases, by Ras and Rho family GTPases and by Gβγ subunits from heterotrimeric G-proteins [200]. Since monomeric p110 is unstable and is rapidly degraded, activation of p85α/p110α does not involve the complex dissociation but would rather depend on conformational changes [201,202]. Tax1 targets p85α and disrupts the p85α/p110α complex leading to increased PI3K activity [203], Akt Ser^473 ^phosphorylation, AP1 activation and ultimately cell proliferation. Consistent with this model, ATL cells display constitutive activation of AP1 [199,204,205].

### 3.6 Smad proteins

Transforming growth factor β (TGFβ) inhibits T cell growth in mid-G1 but can also promote tumorigenesis [206]. TGFβ binds to a heterodimeric complex composed of type I (TβRI) and type II (TβRII) serine/threonine kinase receptors [207]. Upon binding of a TGFβ ligand, TβRII recruits and activates TβRI, which, in turn, phosphorylates downstream targets such as Smad proteins (Smad1-2-3-5-8, receptor activated R-Smad). Common mediator Co-Smad (Smad4) containing complexes then translocate to the nucleus and activate transcription of genes under the control of a Smad-binding element. Signal termination is further mediated by inhibitory Smad (I-Smad) Smad6 and Smad7 [207].

Due to constitutive AP1 activation, ATL cells produce high levels of TGFβ in the sera of HTLV-1 infected patients [208]. TGFβ does not inhibit growth of HTLV-1 infected CD4+ cells but affects CD8-dependent response a mechanism that may impact on immune surveillance [209]. Furthermore, TGFβ stimulates cell surface expression of proteins involved in HTLV binding and fusion (Glut1), leading to enhanced virus transmission and production [210,211].

Tax1 inhibits Smad-dependent signaling, thereby promoting resistance of HTLV-1 infected cells to TGFβ [184,212,213]. This inhibition is mediated by Tax1 interaction with the aminoterminus of Smad2, Smad3, and Smad4 [212]. Through these interactions, Tax1 inhibits complexation and DNA binding of Smad3-Smad4 [184,212]. Furthermore, Tax1 may compete with Smads for the recruitment of CBP/p300 [213].

### 3.7 Cas-L and p130Cas

Proteins belonging to Crk-associated substrate (Cas) family are multiadaptator and scaffold molecules that spatially and temporally control signal transduction downstream of integrins, receptors protein tyrosine kinase, estrogen receptors and GPCRs. Upon binding of a ligand to these receptors, Cas proteins are tyrosine phosphorylated and recruit adaptors and effectors (such as small GTPase) to activate downstream targets such as JNK and ERK. As a result, Cas proteins regulate cell survival, apoptosis and migration. Furthermore, deregulation of Cas functions has been linked to cell transformation, invasion and cancer [214].

Among Cas proteins, Tax1 associates with p130Cas and CasL (lymphocyte type) [215]. CasL, which is preferentially expressed in lymphocytes [216], is phosphorylated and over-expressed in Tax1-expressing cells, in Tax1-transgenic mice as well as in primary lymphocytes isolated from ATL patients [215,217]. The Tax1 and CasL interplay results in enhanced motility of Tax1-expressing lymphocytes in response to fibronectin and CD3 [215]. Since CasL also participates in RhoGTPase activation, Tax1 could interconnect cytoskeleton proteins, stimulate cytoskeleton rearrangement and enhance the motility of leukemic cells.

### 3.8 Global effects of Tax1-mediated deregulation of cell signaling pathways

**Figure 2:**
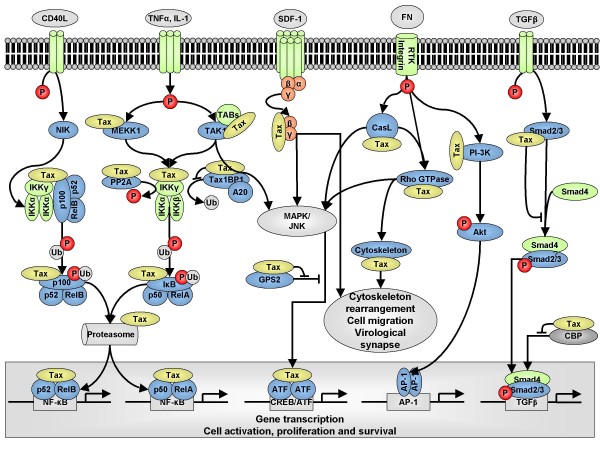
**Overview of cell signaling proteins targeted by Tax1**. Tax1 interacts with components of several signaling pathways (MAPK, JNK, NF-κB, AP-1 and TGF-β) and promotes cellular activation, proliferation, cytoskeleton rearrangement, cell migration and formation of the virological synapse.

As schematized on figure 2, Tax1 interactions with a series of components of several signaling pathways (MAPK, JNK, NF-κB, G proteins, AP1 and TGFβ) affect multiple cellular processes among which cellular activation, proliferation, cytoskeleton rearrangement, cell migration and formation of the virological synapse.

## 4 Interaction of Tax1 with cell cycle associated proteins

### 4.1 Cyclin D-CDK4/6 complexes, Rb and CDK inhibitors

**Figure 3:**
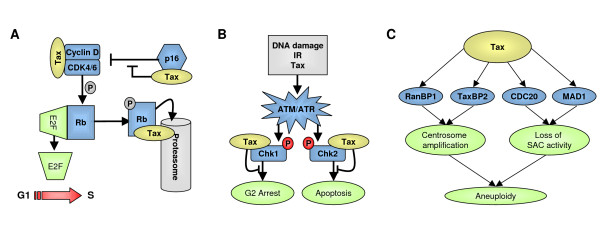
**Effect of Tax1 on cell cycle progression**. Through a series of interactions with cell-cycle associated proteins, Tax1 accelerates G1/S transition (**A**), attenuates Chk1/2 activity (**B**), induces supernumerary centrosomes and impedes SAC (spindle assembly checkpoint) activity (**C**).

Cell cycle progression is a tightly regulated process controlled by cyclins associated with cyclin-dependent kinases (CDK). Cyclins D and E cooperate with CDK4/6 and CDK2 to mediate passage through G1 phase and G1/S transition, respectively [218]. Cyclin D-CDK4/6 and Cyclin E-CDK2 complexes target the Rb retinoblastoma protein (Figure 3). In its hypophosphorylated form, Rb is bound to the transcription factor E2F1, and upon phosphorylation, Rb frees E2F1, which activates transcription of genes required for transition from G1 to S. G1/S progression can be inhibited by CDK inhibitors (CDKI) such as p15^INK4b^, p16^INK4a^, p18^INK4c ^and p19^INK4d ^by preventing cyclin D/CDK4/6 complex formation. Tax1 reprograms cell cycle progression, particularly at G1/S transition, through different mechanisms pertaining to transcriptional activation or repression, post-translational modifications and protein-protein interactions [219,220].

Tax1 is able to interact with cyclins-D1, -D2 and -D3 as well as with CDK4 and CDK6 but not with CDK1 or CDK2 [221-224]. Through these interactions, Tax1 stabilizes the cyclin D2/CDK4 complex and enhances its kinase activity, leading to hyperphosphorylation of retinoblastoma protein (Rb). Tax1 also associates with p15^INK4b ^and p16^INK4a ^and counteracts their inhibitory activity of CDK4 [225-228]. Finally, Tax1 binds to and targets Rb for proteosomal degradation [229]. Consistently, HTLV-1 infected cell lines and freshly isolated ATL cells display decreased levels of Rb protein.

Figure 3A illustrates Tax1 interactions with components of the cyclin D/CDK complexes and provides a mechanistic model for increased G1-S phase transition efficiency as well as the accelerated cell proliferation measured *in vivo *[230,231].

### 4.2 DNA repair pathway associated proteins

DNA insults and replication stress activate the DNA damage response (DDR) pathway in S and G2/M phases of the cell cycle. Activation of the DDR pathway leads to cell cycle delay or even apoptosis of severely damaged cells, and activates the DNA repair pathway. ATM (Ataxiatelangiectasia mutated) and ATR (ATM-Rad3) proteins and their respective downstream targets Chk2 (checkpoint kinase 2) and Chk1 (checkpoint kinase 1) proteins play a central role in the DDR pathway [232]. In mammals, Chk1 and Chk2 regulate Cdc25, Wee1 and p53 that ultimately inactivate CDKs which inhibit cell-cycle progression. Double-strand breaks usually activate the ATM/Chk2-dependent pathway whereas ATR/Chk1 responds to a wide variety of lesions and replication blocks [233,234].

Tax1-expressing and ATL cells display DNA damages suggesting that Tax1 abrogates cellular checkpoint and DNA repair [235-237]. Tax1 binds Rad51 [130] and DNA Topoisomerase 1 [135] that are both directly involved in DNA repair processes [232,238]. Moreover, Tax1 associates and colocalizes with Chk1 and Chk2 proteins [239-242]. *De novo *Tax1 expression causes phosphorylation of Chk2 resulting in accumulation of cells in S-G2/M [239,243]. However, upon gamma irradiation, Tax1 inhibits Chk1 and Chk2 kinase activities and attenuates G2/M arrest and apoptosis, respectively [240,241]. Tax1 thus activates and represses checkpoint controls depending on the experimental conditions (figure 3B). In fact, Tax1 sequesters phosphorylated Chk2 within chromatin after gamma irradiation-induced DNA-damage [242]. Tax1 thereby impedes phosphorylated Chk2 chromatin egress, a mechanism required for further signal amplification and transmission [244]. Tax1 thus targets multiple components of DNA damage repair pathway and promotes DNA abnormalities.

### 4.3 Centrosome associated proteins and spindle assembly checkpoint

One of the hallmarks of Tax1-expressing cells particularly in ATL is chromosomal instability and severe aneuploidy [235], suggesting that mechanisms monitoring chromosomal segregation during mitosis are subverted by Tax1. Tax1 interacts with 4 proteins involved in centrosome amplification or in mitotic spindle assembly checkpoint (SAC) (Figure 3C).

#### 4.3.1 RanBP1 and Tax1BP2

The presence of two centrosomes at mitosis is crucial for formation of bipolar mitotic spindles and correct chromosome segregation. Multipolar mitosis which happens when more than two centrosomes emerge in one cell is a possible cause of aneuploidy in solid tumors and leukemias [245]. Supernumerary centrosomes are observed in approximately 30% of ATL cells [246-248]. Tax1 colocalizes with the centrosome during mitosis and causes centrosome amplification through physical interaction with Ran/Ran Binding protein 1 (RanBP1) and Tax1BP2 [249,250]. RanBP1 is involved in the Ran GTP cycle that controls microtubule nucleation and/or stabilization and centrosome cohesion during mitosis [251,252]. Centrosome fragmentation requires direct Tax1/RanBP1 interaction and Tax1's ability to transactivate NF-κB. Tax1BP2 is thought to act as an intrinsic block to centrosome overreplication [253]. Consistently, overexpression of Tax1BP2 abolishes Tax1-induced centrosome amplification. On the other hand, a Tax1 mutant unable to bind to Tax1BP2 is impaired in centrosome overreplication [250].

#### 4.3.2 Mad1 and cdc20

In eukaryotes, the mitotic spindle assembly checkpoint (SAC) monitors the fidelity of chromosome segregation [254]. SAC functioning requires complex formation between Mad1-2-3 and Bub1-2-3 proteins that arrest mitosis in response to microtubule damage [255]. At the molecular level, SAC activation involves formation of inhibitory complexes between Mad2 and/or Mad3/BubR1 and Cdc20, preventing Cdc20 from activating the anaphase promoting complex/cyclosome (APC/C). APC/C is active during mitosis where it mediates ubiquitination and degradation of an inhibitory chaperone of separase called securin. Once liberated from its inhibitor, separase triggers anaphase by hydrolysing cohesin leading to subsequent separation of sister chromatin. Furthermore, APC/C regulates the degradation of mitotic cyclin, activates CDK1 and, ultimately, promotes mitotic exit [256].

Through physical interactions with Mad1 and Cdc20, Tax1 subverts activation of SAC and APC/C. Tax1 inhibits Mad1 homodimerization, a process that is required for formation of a inhibitory complex between Mad2 and Cdc20 [257-259]. Consistently, ATL cells exhibit a defect in the mitotic spindle assembly checkpoint [257]. On the other hand, Tax1 associates with and activates Cdc20-associated APC/C leading to unscheduled degradation of securin and cyclin B1, a delay or failure in mitotic entry and progression, and faulty chromosome transmission [260,261]. Tax1-induced premature activation of APC/C provokes permanent G1 arrest and senescence [262,263]. Finally, coexpression of Tax1 and securin enhances chromosomal instability and favors cell transformation *in vitro *and *in vivo *[264].

## 5 Interaction of Tax1 with PDZ-containing proteins

The PSD-95/*Drosophila *Discs Large/Zona Occludens-I (PDZ) domain containing proteins form signaling complexes at the inner surface of the cell membrane and are involved in a very broad range of functions like cell signaling, adhesion, tight-junction integrity, molecular scaffolding for protein complexes and tumor suppression [265-267]. Numerous PDZ proteins have been shown to form a complex with Tax1 owing to its PDZ binding motif (PBM) located in the C-terminus (ETEV) [268]: Pro-IL16 (precursor of interleukin 16) [269], hDLG (*Drosophila *Discs Large) [270,271], PSD-95, beta-syntrophin, lin-7 [268], Tip1 (Tax1 Interaction protein 1) [272], MAGI3 (Membrane Associated Guanylate kinase with inverted orientation 3) [273], hTid1 [274] and hScrib [275]. Interaction of Tax1 with these PDZ proteins frequently leads to their delocalization [273,275,276]. Functionally, PDZ proteins such as hTid1 and hScrib participate to Tax1-mediated activation of NF-κB and NFAT pathways, respectively [274,275].

A Tax1-binding protein, hDLG, has been particularly studied owing to its ability to act as a tumor suppressor. hDLG acts downstream of the Wnt/frizzled pathway and binds to the adenomatous polyposis complex (APC) which mediates cell cycle progression [277,278]. APC-hDLG complex formation negatively regulates G1 to S transition and plays an important role in transducing the APC cell cycle blocking signal [277]. Besides, hDLG is also involved in maintenance and modulation of T cell polarity [279]. Through PBM/PDZ domain interaction, Tax1 induces hyperphosphorylation of hDLG, affects its localization [276] and prevents its binding to APC [271]. Interestingly, hDLG inactivation increases the ability of Tax1 to transform a mouse T-cell line [280].

The Tax1 PBM is critically involved in transformation of rat fibroblasts and IL2 independent growth of mouse lymphocytes [276,281] and to promote virus-mediated T-cell proliferation *in vitro *and persistence *in vivo *[282]. In contrast, HTLV-2 Tax2 protein which does not harbor a PBM has a lower transforming activity than Tax1 [283].

## 6 Tax1 interaction with nuclear pore and secretory pathway proteins

Tax1 shuttles between the cytoplasm and the nucleus by virtue of a nuclear localization sequence (NLS) and a nuclear export signal (NES) [284-286]. In the nucleus, Tax1 is primarily located in interchromatin granules or spliceosomal speckles [141]. In the cytoplasm, Tax1 localizes to organelles associated with the cellular secretory process including the endoplasmic reticulum and Golgi complex [192,287]. Tax1 is also secreted in the supernatant of HTLV-1 infected cells isolated from HAM-TSP patients [287-289] and may behave as an extracellular cytokine. Tax1 shuttling is mediated through interaction with proteins involved in nuclear import, cytoplasmic export and secretory pathways [289-293].

### 6.1 Nucleoporins

Nucleoporins of the nuclear pore complex (NPC) form a channel spanning the double lipid bilayer of the nuclear envelope. Nuclear pore complexes allow passive diffusion of ions and small proteins but translocation of cargoes larger than 40 kDa generally requires specific transport proteins [294]. Import of cargo proteins containing a classical NLS is mediated by the importin α/β dimer and requires metabolic energy which is provided by Ran GTP [295]. In contrast, carrier-independent translocation of proteins into the nucleus is energy independent and requires direct interactions with nucleoporins [295].

Nuclear import and export of Tax1 are both carrier and energy independent but relies on the interaction between Tax1 and the p62 nucleoporin [290]. This interaction is mediated by the aminoterminal zinc-finger motif of Tax1. Consistently, mutations within this motif abolishes Tax1 interaction with p62 and nuclear import [290].

### 6.2 Proteins involved in Tax1 nuclear export and secretion

Proteins containing a NES domain like Tax1 are expected to interact with the chromosome region maintenance 1 protein (CRM1), a member of the importin β family [296]. Under stress conditions (i.e. UV irradiation), Tax1 interacts with CRM1 and is exported outside of the nucleus, a mechanism that is inhibited by leptomycin B [291,292]. In the absence of stress however, leptomycin B does not alter subcellular distribution of Tax1 [286], suggesting that Tax1 is not exclusively exported through the CRM1 pathway.

Tax1 nucleo-cytoplasmic shuttling and secretion is directed by associations with proteins involved in nuclear export (calreticulin, RanBP2, p97), in ER to Golgi transport (the coat proteins (COP) βCOP and COPII) and in movement from Golgi to plasma membrane (secretory carrier membrane protein 23 (SNAP23), secretory carrier membrane protein 1 and 2 (SCAMP1, SCAMP2)) [289,293,297]. Calreticulin, which is overexpressed in HTLV-1 infected cells, functions similarly to CRM1 by transporting proteins via NES interactions [293,298]. Tax1 secretion involves a secretory signal located in the C-terminal domain and requires interaction with SNAP23, SCAMP1 and COPII [289].

Tax1 thus targets different cellular factors involved in protein transport to shuttle between nucleus, cytoplasm and extracellular environment.

## 7 Binding domains in Tax1

**Figure 4:**
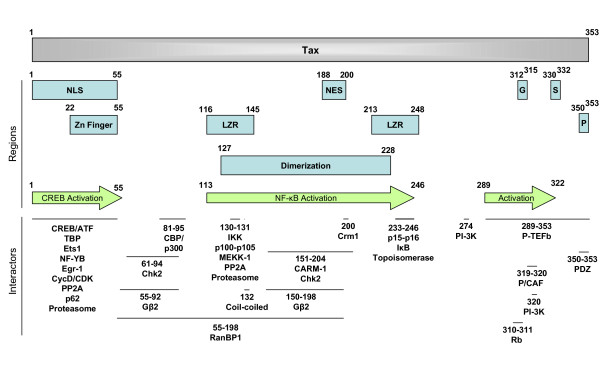
**Functional regions of Tax1 and interaction domains**. NLS (nuclear localization sequence), NES (nuclear export sequence), G (Golgi localization motif), S (secretion motif), LZR (leucine-zipper-like region), P (PDZ binding domain). Adapted from [130].

To interact with such a broad range of cellular targets, Tax1 contains multiple protein-binding domains (Figure 4). Among these, the N-terminal zinc finger motif associates with transcription factors (CREB/ATF [299], TBP [90], Ets1 [62], NF-YB [82], Egr1 [85]), cyclins [221], nucleoproteins (p62) [290], proteasome subunits [163] and phosphatase PP2A [168]. Mutations within this zinc finger affects Tax1-mediated CREB transactivation as well as subcellular localization due to the presence of a NLS [284]. A domain encompassing residues 55 to 95 regulate interaction of Tax1 with CBP/p300, Chk2 and Gβ2 [102,241,300]. The middle of Tax1 harbors a region required for dimerization, two leucine zipper-like motifs (aa 116–145 and 213–248) [39,301,302] and a NES sequence [291]. Substitutions within the first leucine zipper (such as T130A and L131S in mutant M22) affect Tax1 interactions with NF-κB [157,301], proteasome subunits [163] and PP2A [168]. Another mutation (S132A) abolishes Tax1 binding to coil-coiled domain containing proteins [303] (i.e. Mad1, Tax1BP1, Tax1BP2 and GPS2). A region located between the two leucine zippers is required for interaction with CARM-1, Chk2 and Gβ2 [127,241,300]. Amino acids 233–246, located within the second leucine zipper regulates Tax1 association with p15^INK4b ^[228], p16^INK4a ^[226], DNA topoisomerase [135] and IκBγ [161]. Consistently, the central region of Tax1 is indeed involved in NF-κB activation. Finally, the carboxyterminal region of Tax1 contains an activation domain (residues 289–332) [304] as well as motifs required for Tax1 localization within the Golgi (residues 312–315) and secretion (residues 330–332) [297]. The carboxyterminal domain is involved in Tax1 binding to Rb [229], PI3K [199], P/CAF [102], P-TEFb [138] and PDZ containing-proteins [268]. In particular, Tax1 mutant M47 (L319R, L320S) is impaired for interaction with P/CAF[102].

## 8 Conclusion

**Figure 5:**
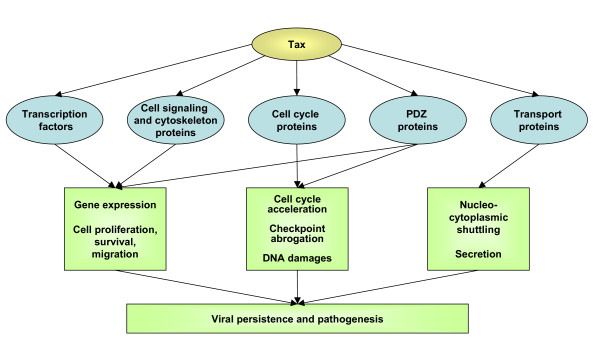
Overview of the Tax1 interactome.

The most intriguing point relating to the Tax1 interactome is the very high number of cellular proteins to which this viral oncogene is able to interact. Today, about 100 Tax1-binding proteins are identified (Table 1) and this number is permanently growing (see  for regular updates). Is it possible that a single protein modulates such a wide variety of functions? Are these interactions all relevant for the viral life cycle or pathogenesis? As schematized on Figure 5, the vast majority of these interactions contributes to viral or cellular gene expression and promotes infected cell proliferation or survival, required for maintaining viral load *in vivo *[231,305]. On the other hand, checkpoint abrogation allows proliferation of cells with DNA lesions and progressive accumulation of chromosomal abnormalities as frequently observed in ATL [220]. Even if one might entertain doubts about the biological relevance of some Tax1 partners, the Tax1 interactome as a whole likely contributes to the viral life cycle as well as to development of pathogenesis.

**Table 1:** Cellular proteins interacting with Tax1

**Transcription and translation**	**Cell signaling**
	
ATF1 [28]	CasL [215]
ATF2 [30]	Cdc42 [130]
ATF3 [27]	Gap1m [130]
ATF4 [29]	GPS2 [183]
ATFx [42]	Gβ2 [300]
BAF155 [130]	IKKα [159]
BAF53 [130]	IKKβ [159]
BAF57 [130]	IKKγ [156]
BRG1 [130]	IκBα [162]
C/EBPβ [81]	IκBγ [161]
CARM1 [127]	MEKK1 [157]
CREB [26]	p100 [317]
c-Rel [75]	p105 [163]
CREM [28]	p130Cas [215]
Cyclin T1 [138]	p85α [199]
DNA topoisomerase I [135]	PP2A [168]
Egr1 [85]	Rac [130]
Elk1 [63]	RhoA [130]
Ets1 [62]	Smad2 [213]
HDAC1 [119]	Smad3 [213]
Int6 [149]	Smad4 [213]
JMJD2A [125]	TAB2 [181]
MBD2 [123]	TAK1 [158]
MSX2 [89]	Tax1BP1 [171]
NF-YB [82]	Ubc13 [170]
NRF1 [88]	
p/CAF [101]	
p300/CBP [100]	
	
p50 [61]	**PDZ proteins**
	
p52 [74]	beta-syntrophin [268]
RelA [75]	hDLG [270]
RPL6 [318]	hScrib [275]
Sap1 [63]	hTid1 [274]
Sc35 [141]	lin7 [268]
SMRT [122]	MAGI3 [273]
Sp1 [62]	Pro-IL16 [269]
Spi-1 [81]	PSD95 [268]
SRF [59]	Tip1 [272]
SUV39H1 [124]	
TAF_II_28 [91]	
Tax1BP1 [144]	
	
TBP [90]	**Transport**
	
TF_II_A [92]	Calreticulin [289]
TORC1 [44]	COPII [289]
TORC2 [44]	CRM1 [291]
TORC3 [43]	p62 [290]
TRBP [150]	p97 [289]
TTP [148]	RanBP2 [289]
XBP1 [31]	SCAMP1 [289]
	SCAMP2 [289]
	SNAP23 [289]
	
**Cell cycle and DNA repair**	βCOP [289]
	
CDC20 [260]	
CDK4[221]	
CDK6 [221]	
	
Chk1 [240]	**Cytoskeleton**
	
Chk2 [239]	Actin [130]
Cyclin D1 [224]	Annexin [130]
Cyclin D2 [221]	Cytokeratin [190]
Cyclin D3 [224]	Gelsolin [130]
Mad1 [258]	α-internexin [29]
p15^INK4b ^[228]	γ-tubulin [191]
p16^INK4a ^[225]	
Rad51 [130]	
RanBP1 [249]	
	
Rb [229]	**Proteasome**
	
Tax1BP2 [250]	HC9 [163]
Topoisomerase 1 [135]	HSN3 [163]

Other viral oncogenes such as Kaposi's sarcoma-associated herpesvirus-encoded LANA and adenovirus E1A also interact with numbers of cellular proteins (e.g. more than 40 for E1A and 100 for LANA) [306,307]. Interestingly, some of these proteins are targeted both by Tax1 and E1A (such as ATF, CBP, p300 or Smad), indicating that similar signaling pathways are involved in distinct viral systems to achieve cell transformation. In particular, Tax1 and E1A share common properties that include regulation of transcriptional activation, chromatin remodeling, interference with p53 activity, regulation of proteasome function and cooperation with Ras in cell transformation [308].

How are these different activities controlled temporarily and spatially? Additional studies are definitely required to address this point. Currently, Tax1 is known to shuttle between cytoplasm and nucleus, to form intranuclear speckles along with a series of cellular proteins (e.g. NF-κB factors [309], sc35 [141] and chk2 [239]) and to target specialized structures such as the centrosome [249,250]. Moreover, Tax1 localisation and protein interactions are altered under stress conditions [291,292].

**Figure 6:**
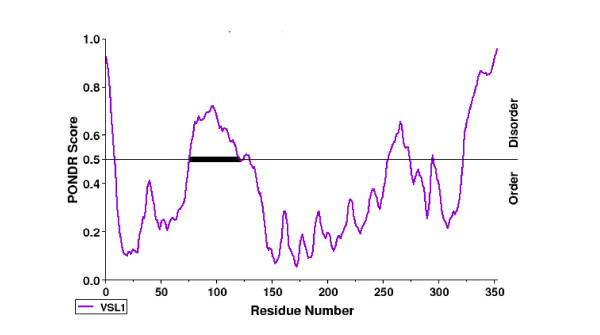
**Identification of disordered regions in Tax1 according to the VSL1 algorithm**. (PONDR^®^, ). A domain encompassing residues 76 to 121 (black bar) corresponds to a long disordered region within Tax1.

Despite numbers of attempts, Tax1 3-D crystallographic structure is intriguingly still unsolved suggesting that Tax1 adopts a rather undefined conformation. In this context, the concept of intrinsically disordered proteins (IDP) has recently emerged [310]. IDPs contrast to "ordered" proteins that fold into a unique and structured state, which represents a kinetically accessible and energetically favorable conformation. IDP proteins contain one or multiple disordered regions that exist as dynamic ensembles in which atom positions and backbone Ramachandran angles vary significantly with no specific equilibrium values [310]. The presence of short (< 30 residues) and long (> 30 residues) ID regions confer conformationnal flexibility thereby facilitating post-translational modifications and enabling a protein to functionally interact with many cellular partners [310,311]. Consistently, IDPs are frequently highly connected 'hubs' in the protein-protein networks [311-313]. In fact, Tax1 contains many proline (n = 40), serine (n = 25) and glycine (n = 25) residues that are known to promote disorder [310]. According to the VSL1 prediction programme (PONDR^®^, ), Tax1 contains multiple ID regions (n = 6) (Figure 6). In particular, Tax1 contains a long disordered region (spanning amino-acids 76 to 121), in contrast to the well structured capsid (p24), transmembrane (gp21) and surface (gp46) proteins (data not shown). Interestingly, other viral oncogenes such as HPV E6 and E7 are also predicted to contain significant intrinsic disorder [314].

On the other hand, Tax1 is modified by phosphorylation, ubiquitination and sumoylation that potentially modulate its functions, localisation and interactions [76,315]. Tax1 also contains 8 cysteines that may form disulfite bonds or coordinate zinc ions and 48 leucines that are considered as order-promoting residues [310]. Tax1 thus appears as a flexible structure formed by a series of small modular domains that are relatively independent of surrounding sequences and that permits wide conformational changes depending upon its subcellular environment.

We propose that, similarly to the hubs, the ID-based structure of Tax1 allows a wide variety of conformational changes enabling binding diversity and recognition of differently shaped protein partners. Flexible accommodation at various binding interfaces would then allow interaction of more structured domains such as the Tax1 zinc finger and leucine containing helices. This hypothetical model provides a rationale to the very broad range of Tax1 interacting proteins identified so far.

In conclusion, the Tax1 interactome network with the associated biochemical studies reported here provides a molecular basis for understanding viral persistence and pathogenesis, paving the way for the design of compounds to antagonize its ability to mediate cell transformation.

## Competing interests

The authors declare that they have no competing interests.

## Authors' contributions

MB and LW collected data from the literature and wrote the paper, JT, SL and RK suggested comments, FD provided technical help. All authors read and approved the final manuscript.
